# Expression of Par3 polarity protein correlates with poor prognosis in ovarian cancer

**DOI:** 10.1186/s12885-016-2929-2

**Published:** 2016-11-17

**Authors:** Hiroe Nakamura, Kazunori Nagasaka, Kei Kawana, Ayumi Taguchi, Yuriko Uehara, Mitsuyo Yoshida, Masakazu Sato, Haruka Nishida, Asaha Fujimoto, Tomoko Inoue, Katsuyuki Adachi, Takeshi Nagamatsu, Takahide Arimoto, Katsutoshi Oda, Yutaka Osuga, Tomoyuki Fujii

**Affiliations:** Department of Obstetrics and Gynecology, Faculty of Medicine, The University of Tokyo, Tokyo, 113-8655 Japan

**Keywords:** Ovarian cancer, Par3, STAT3, IL-6

## Abstract

**Background:**

Previous studies have shown that the cell polarity protein partitioning defective 3 (Par3) plays an essential role in the formation of tight junctions and definition of apical-basal polarity. Aberrant function of this protein has been reported to be involved in epithelial–mesenchymal transition (EMT) and cancer invasion. The aim of this study was to examine the functional mechanism of Par3 in ovarian cancer.

**Methods:**

First, we investigated the association between Par3 expression level and survival of 50 ovarian cancer patients. Next, we conducted an in vitro analysis of ovarian cancer cell lines, focusing on the cell line JHOC5, to investigate Par3 function. To investigate the function of Par3 in invasion, the IL-6/STAT3 pathway was analyzed upon Par3 knockdown with siRNA. The effect of siRNA treatment was assessed by qPCR, ELISA, and western blotting. Invasiveness and cell proliferation following treatment with siRNA against Par3 were investigated using Matrigel chamber, wound healing, and cell proliferation assays.

**Results:**

Expression array data for ovarian cancer patient samples revealed low Par3 expression was significantly associated with good prognosis. Univariate analysis of clinicopathological factors revealed significant association between high Par3 levels and peritoneal dissemination at the time of diagnosis. Knockdown of Par3 in JHOC5 cells suppressed cell invasiveness, migration, and cell proliferation with deregulation of IL-6/STAT3 activity.

**Conclusion:**

Taken together, these results suggest that Par3 expression is likely involved in ovarian cancer progression, especially in peritoneal metastasis. The underlying mechanism may be that Par3 modulates IL-6 /STAT3 signaling. Here, we propose that the expression of Par3 in ovarian cancer may control disease outcome.

**Electronic supplementary material:**

The online version of this article (doi:10.1186/s12885-016-2929-2) contains supplementary material, which is available to authorized users.

## Background

Ovarian cancer is the fifth most frequent cause of cancer-related deaths among women worldwide [1]. The estimated annual incidence of ovarian cancer is approximately 225,000 women, resulting in 140,200 deaths per year [2]. The prognosis for advanced disease has not improved significantly in more than two decades [3], suggesting that a better understanding of progression and metastasis mechanisms of ovarian cancer is critical for determining new ways to prevent, diagnose, and treat this disease.

Loss of polarity and epithelial cell organization is a hallmark of carcinoma invasion and metastasis [4–6]. Loss of polarity is considered the initial step of the epithelial mesenchymal transition (EMT), which is characterized by the loss of cell-cell adhesion and apical-basal cell polarity, along with increased cell motility [4, 7–11].

Three major complexes involved in regulating epithelial cell apical-basal polarity have been described: the Crumbs complex and Par complex, which are found apically, and the Scribble complex, located at the basolateral membrane [4, 12, 13]. Among these three polarity complexes, the Par complex is the best-studied [5, 14–16]. The Par complex consists of three proteins: Par3, Par6, and aPKC (atypical protein kinase C). Par3 is essential for the delivery of aPKC to the apical surface through binding of Par3 to the adaptor protein Par6, which forms a constitutive complex with aPKC [17, 18]. This complex is involved not only in the formation of apical-basal polarity, but also in cell proliferation, migration, and asymmetric cell division [19–21].

Recent studies have identified the Par complex as an important regulator in tumorigenesis and metastasis [22–29]. However, the involvement of Par3 in this process may be highly context-dependent. Genome-wide screening for microdeletions revealed that the region containing the Par3 gene (*PARD3*) is deleted in lung, head and neck, and esophageal squamous cell carcinoma cell lines [29, 30]. In breast, esophageal, and lung cancers, Par3 seems to act as a tumor suppressor [22, 23, 25], whilst in clear-cell renal carcinoma, Par3 overexpression is associated with poor prognosis [27, 28]. In skin cancer, Par3 may act as a tumor suppressor or tumor promoter depending on the tumor type [24]. The detailed mechanism of how Par3 is involved in tumorigenesis and invasion may depend on tumor type and is still to be elucidated. The Rac1/JNK proliferation pathway and the IL-6/STAT3 pathway may be key for understanding the functions of Par3 in promoting cancer growth [22, 25, 31, 32].

In ovarian cancer, overexpression of aPKC is known to be associated with poor prognosis [33, 34] but there has been little research on the function of Par3 in pathogenesis. The goal of this study was to analyze the functions of Par3 and to investigate the Par3-related pathways that might be relevant to the clinical outcome and to understanding the pathogenesis of epithelial ovarian cancer.

## Methods

### Antibodies and reagents

The following antibodies were used at the dilution indicated. For western blotting: anti-Par3 Millipore #07-330 (1:500) was purchased from Merck Millipore (Darmstadt, Germany); anti-alpha Tubulin sc-8035 (1:500), anti-Vimentin (V9) sc-6260 (1:500), and anti-CD71(TFR) (3B82A1):sc-32272 (1:500) were purchased from Santa Cruz Biotechnology (Texas, USA); anti-total Stat3 124H6 CS#9139 (1:1000) and anti-phospho Stat3 (Tyr705) (D3A7) CS#9145 (1:1000) were purchased from Cell Signaling Technologies (Massachusetts, USA); and anti-E-Cadherin BD 610181 (1:500) was purchased from BD (California, USA). For immunofluorescent analysis: anti-Par3 ab64646 (1:100) was purchased from Abcam (Massachusetts, USA) and anti-phospho Stat3 (Tyr705) (D3A7) CS#9145 (1:100) was purchased from Cell Signaling Technologies. The STAT3 Inhibitor S3I-201 (SC-204304) was purchased from Santa Cruz Biotechnology.

### Cell culture

Ovarian cancer cell lines were maintained in the following media supplemented with 10% fetal bovine serum (FBS, Life Technologies, California, USA) and antibiotics (Antibiotic-Antimycotic Mixed Stock Solution, Nacalai Tesque, Kyoto Japan). JHOC5 was maintained in Dulbecco's modified Eagle medium: Nutrient Mixture F-12 (DMEM/F-12, Life Technologies, California, USA). HaCaT and SKOV3 were maintained in Dulbecco’s modified Eagle medium (DMEM, Wako, Osaka Japan). OVISE, OVTOKO, and TOV21 were maintained in RPMI (Wako, Osaka Japan). RMG1 was maintained in F-12 (Life Technologies). All cells were grown in a humidified tissue culture incubator at 37 °C in 5% CO_2_.

### Transfections

Small Interfering RNAs were transfected using Stealth RNAi against PAR3 (HSS125534, HSS183488, HSS183489), STAT3 (HSS186130, HSS186131, HSS110279), or non-targeting siRNA (Stealth RNAi siRNA Negative Control, Med GC, Life Technologies) as a control. When cells were 60–70% confluent, transfections were performed using Lipofectamine RNAiMAX (Life Technologies), Opti-MEM Reduced Serum Medium (Life Technologies), and a final concentration of 20 nmol/L siRNAs, according to the manufacturer’s instructions. After 5 h of incubation, the transfection medium was changed to normal culture medium without antibiotics. The cells were incubated for 48 h and then subjected to experimentation. Transfection experiments were repeated at least 3 times.

Wild type myc-tagged Par3 was transfected using Effectene Transfection Reagent (Qiagen, Hilden, Germany) according to the manufacturer’s instructions. The plasmid was kindly provided by Dr. Vjeko Tomaić (International Centre for Genetic Engineering and Biotechnology, Italy).

### Immunoblotting

Cells were lysed by incubation in Lysis buffer (Cell Signaling Technologies #9803) containing protease inhibitor cocktail (Nacalai Tesque, Kyoto Japan) and phosphatase inhibitor cocktail (Roche, Basel, Switzerland) on ice for 5 min. Lysates were then sonicated briefly and centrifuged at 14,000 rpm at 4 °C for 10 min. The supernatants were analyzed as follows: for SDS-PAGE, 20 μg of protein was loaded in each well. For immunoblotting, 0.45 μm PVDF membranes (Merck Millipore) were used. The membranes were blocked in 5% milk/TBS-T (TBS containing 0.1% Tween-20) for 1 h at 20–25 °C followed by incubation with the appropriate primary antibody diluted in 5% milk/TBS-T or 5% BSA/TBS-T for the appropriate time according to the manufacturer’s instructions. After several washes with TBS-T, membranes were incubated with the appropriate HRP-conjugated secondary antibody in 5% milk/TBS-T at 20–25 °C for 1 h. Blots were developed using Immobilon Western Chemiluminescent HRP substrate (Merck Millipore) according to the manufacturer's instructions.

### Subcellular fractionation assays

To obtain cytoplasmic, nuclear, and membrane fractions from the cells, a subcellular fractionation assay was performed using the Calbiochem ProteoExtract Fractionation Kit (Merck Millipore) according to the manufacturer's instructions. To inhibit phosphatase activity during lysate preparation, phosphatase inhibitor cocktail (Roche, Basel, Switzerland) was used.

### Reverse transcription and qPCR

Total RNA was extracted from the cell lines using a Cultured Cell Total RNA Purification Mini Kit (FAVORGEN, Ping Tung, Taiwan) followed by reverse transcription using ReverTra Ace qPCR RT Master Mix (Toyobo, Osaka, Japan) according to manufacturer’s instructions. cDNA was amplified for 40 cycles in a LightCycler 480 instrument (Roche, Basel, Switzerland) using LightCycler 480 SYBR Green I Master reagent (Roche). The primer sets used for qPCR are: for Par3, 5′-CGCTTGGAACATGGAGATGG-3 and 5′-ATCTCTGGGCTCTGGGTACC-3, for GAPDH, 5′-GAAAGGTGAAGGTCGGAGTC-3 and 5′-GAAGATGGTGATGGGATTTC-3. mRNA levels of each gene were normalized to GAPDH mRNA as an internal standard. Expression levels were calculated by the comparative Ct method using GAPDH as the endogenous reference gene.

### Invasion assay

JHOC5 cells were treated with siRNA against Par3, STAT3, or negative control siRNAs, and then incubated for 48 h. Cells were trypsinized and dissociated from dishes, then used for invasion assays. Matrigel invasion assays were performed using 24-well BioCoat Matrigel invasion chambers (Corning international, NY USA) according to the manufacturer’s instructions. Briefly, lower chambers were filled with 750 μL of DMEM/F12 with 10% FBS and chemical reagents. Cells in 500 μL of FBS-free medium were applied to the upper chamber and incubated for 24 h. After incubation, the cells remaining in the upper chamber were removed with cotton swabs and the cells that had invaded through the Matrigel were stained with a Diff Quik staining kit (Sysmex, Hyogo, Japan). Matrigel membranes were cut from the upper chamber and placed on microscope slides, then observed with an optical microscope.

### Cell migration assay

Cells were seeded onto 6-well culture plates and grown as a monolayer until 100% confluent. A scratch was made on a uniform layer of cells using a sterile micropipette tip followed by one PBS wash to remove debris. Photographs of the same area of the wound were taken after 8 h (for siPar3) or 14 h (for siSTAT3), to measure the width of the wound using a fluorescence microscope (BZ-9000; Keyence, Osaka, Japan).

### Immunofluorescence and confocal microscopy

For immunofluorescence, cells were grown on glass coverslips until 80% confluence and fixed in 4% paraformaldehyde in phosphate-buffered saline (PBS) for 20 min at room temperature. After washing with PBS, the cells were permeabilized in PBS/0.1% Triton X-100 at room temperature for 5 min or 100% methanol at -20 °C for 10 min depending on which primary antibody was being used. Then, the samples were washed extensively in PBS and incubated with the appropriate primary antibody diluted in antibody dilution buffer (PBS/1% BSA/0.3% Triton X-100) for 1 h at room temperature or at 4 °C overnight. After several PBS washes, samples were incubated with the appropriate Alexa Fluor 488- or 548-conjugated secondary antibodies for 1 h at room temperature. After several PBS washes, the coverslips were mounted on glass slides. Cells were visualized using a Zeiss Axiovert 100 M microscope (Zeiss, Milan, Italy) attached to a LSM 510 confocal unit.

### Cell proliferation assay

To analyze the effect of Par3 or STAT3 knockdown on cell proliferation, cell proliferation assays were performed. Five thousand cells were seeded into each well of 96-well plates after 48 h of siRNA transfection. Cell Counting Kit-8 using the tetrazolium salt WST-8 [2-(2-methoxy-4-nitrophenyl)-3-(4-nitrophenyl)-5-(2,4-disulfophenyl)-2H-tetrazolium, monosodium salt (Dojindo, Tokyo, Japan) was used to quantify the number of cells by monitoring the changes in the absorbance at 450 nm, which were normalized relative to the absorbance of cells transfected with non-targeting siRNA.

### Enzyme-linked immunosorbent assay (ELISA)

To analyze the effect of Par3 knockdown on IL-6 levels, an ELISA for IL-6 was performed. Five thousand cells were seeded into each well of 96-well plates followed by incubation for 24 h. Cells were then transfected with siRNA against Par3, and 48 h later, the supernatant was collected for ELISA analysis. Human IL-6 DuoSet ELISA Kit (R&D systems, Minnesota, USA) was used for IL6 detection according to the manufacturer’s instruction.

### Expression array and statistical analysis

Ovarian cancer samples and genomic cDNA were obtained, and expression array analysis was performed as previously described [35]. We use probe set 210094_s_at (GeneChip Human Genome U133 Plus 2.0 Array, Affymetrix, CA, USA) to measure patient *PARD3* mRNA levels. For normalization, we used probe intensity data taken from normal ovarian tissue sample for the probe set 210094_s_at (GeneChip Human Genome U133 Plus 2.0 Array, Affymetrix, Tokyo, Japan) indicating the expression level of *PARD3* mRNA. Then we widened the parameter of normal values by 10% and regarded this value as “intermediate.” Measured values *of PARD3* mRNA above this range were regarded as “high expression,” and below the range were regarded as “low expression.” All patients provided written informed consent for the research use of their samples, and the collection and use of tissues for this study were approved by the Human Genome, Gene Analysis Research Ethics Committee at the University of Tokyo.

Briefly, samples from 50 patients (22 clear-cell carcinomas, 16 serous adenocarcinomas, and 12 endometrioid carcinomas) who underwent primary tumor resection at the University of Tokyo Hospital were used (Table 1). All patients received primary surgery, including hysterectomy, bilateral salpingo-oophorectomy, and omentectomy, together with systematic lymphadenectomy (when mass reduction was completely or optimally achieved). The patients with stage IC–IV received six to eight cycles of adjuvant chemotherapy (paclitaxel and carboplatin). Fresh-frozen tumor samples were embedded in OCT (optimum cutting temperature) compound, and 4-mm thick tissue sections were stained with hematoxylin and eosin. Tissue sections with a high proportion of carcinoma cells (>50%) were reviewed by a pathologist and selected for DNA and total RNA extraction. Genomic DNA was isolated from tumor sections using a QIAamp DNA Mini Kit (Qiagen), according to the manufacturer’s protocol. A Fisher’s exact test was used to evaluate the association between Par3 expression and stage, tumor grade, dissemination, and sites of metastasis. All tests were two-sided and p-values of 0.05 or less were considered statistically significant. Statistical analyses were performed using the JMP12 statistical program (SAS Institute, Cary, NC). Kaplan-Meier plots for progression-free survival (PFS) and overall survival (OS) were plotted and analysis was done using the log-rank test.

**Table 1:** Patient characteristics (*n* = 50)

	No. Patients (*n* = 50)
Age, median (range), yr	57 (32–80)
Follow-up period (m)	59.1 (2–120)
FIGO	
Stage I	25 (50%)
Stage II	4 (8%)
Stage III	12 (24%)
Stage IV	9 (18%)
Histology	
High-grade serous	16 (30%)
Endometrioid	12 (8%)
Clear cell	22 (44%)
Dissemination and metastasis at diagnosis (overlapped, depending on the cases)	
Dissemination	18 (36%)
Lymph node metastasis	12 (24%)
Distant metastasis	3 (0.6%)
Recurrent site (overlapped, depending on the cases)	
Dissemination	17 (34%)
Lymph node metastasis	10 (20%)
Distant metastasis	6 (12%)
Par3 expression	
High	10 (20%)
Intermediate	10 (20%)
Low	30 (60%)

## Results

### Low Par3 expression is associated with good prognosis in ovarian cancer patients

**Fig. 1:**
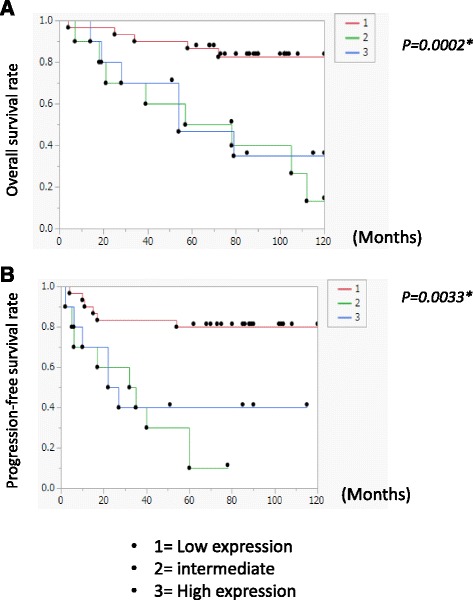
Low Par3 expression is associated with good overall survival and prognosis. We analyzed the duration of response; overall survival (OS), and progression-free survival (PFS) using resected samples from 50 ovarian cancer patients (22 clear-cell carcinomas, 16 serous adenocarcinomas, and 12 endometrioid carcinomas). All patients received primary surgery, including hysterectomy, bilateral salpingo-oophorectomy, and omentectomy, together with systematic lymphadenectomy (when mass reduction was completely or optimally achieved). The patients with stage IC–IV received six to eight cycles of adjuvant chemotherapy (paclitaxel and carboplatin). There were three patients with stage Ia who did not receive any chemotherapy. In this study, the patients with neoadjuvant chemotherapy were excluded. Tissue sections with a high proportion of carcinoma cells (>50%) were reviewed by a pathologist and selected for DNA and total RNA extraction. Kaplan-Meier plots for overall survival (**a**) and progression free survival (**b**) were plotted and analysis was performed using the log-rank test. Low Par3 expression was significantly associated with good prognosis (OS: *P* = 0.002; PFS: *P* = 0.0033)

First, we analyzed the expression microarray data to investigate the relevance of Par3 expression in ovarian cancer prognosis. Samples from 50 ovarian cancer patients (22 clear-cell carcinomas, 16 serous adenocarcinomas, and 12 endometrioid carcinomas) were analyzed. The characteristics and histologic data of study participants are shown in Table 1. In univariate analysis with a hazard ratio and 95% confidence interval, high Par3 expression was significantly associated with advanced stage and peritoneal dissemination at diagnosis, but it was not associated with lymph node metastasis or distant metastasis (Table 2). Moreover, low Par3 expression was strongly associated with good OS and PFS (Fig. 1). These results suggest that cellular Par3 expression may promote peritoneal metastasis in the majority of ovarian cancer patients.

**Table 2:** Correlation between high expression of Par3 and clinical features

Stage	*P* value
Stage I, II *vs* Stage III, IV^a^	*P* = 0.0352*
Dissemination and site of metastasis at diagnosis	
Dissemination	*P* = 0.0381*
Lymph node metastasis	*P* = 0.1469
Distant metastasis	*P* = 0.2914

*P < .05 considered significant

### Par3 is stably expressed in the cytoplasm of JHOC5 cells

**Fig. 2:**
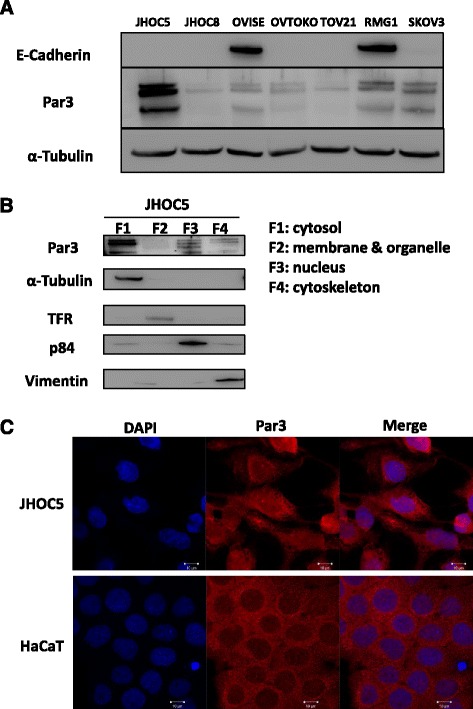
Expression pattern of Par3 in JHOC5 ovarian cancer cells. **a** Par3 protein level in seven ovarian cancer cell lines (JHOC5, JHOC8, OVISE, OVTOKO, TOV21, RMG1, and SKOV3). Semiconfluent cells were harvested for western blotting. Cell lysates were resolved by SDS-PAGE and immunoblotted with an antibody against Par3 or E-Cadherin. Blots are representative of at least three experiments. **b** Subcellular fractionation assay. JHOC5 cells were fractioned into cytosol (F1), membrane (F2), nucleus (F3), and cytoskeleton (F4) pools and Par3 was detected by western blotting. Loading controls used were α-Tubulin, TFR, p84, and vimentin for the cytosol, membrane, nucleus, and cytoskeleton fractions, respectively. **c** Immunofluorescence analysis of Par3 expression in JHOC5 cells and control HaCaT cells. Cells were grown on coverslips and then fixed and stained with the anti-Par3 antibody and DAPI. Scale bar indicates 10 μm

Par3 protein is a tight junction protein that localizes to the membranes of epithelial cells [36, 37]. However, mislocalization of Par3 protein is thought to disrupt normal cell functions including the formation of cell polarity, cell proliferation, and migration [19, 22–26]. First, we analyzed the protein level of Par3 in seven ovarian cancer cell lines, including JHOC5 (clear-cell adenocarcinoma) (Fig. 2a). The level of Par3 expression varied among the ovarian cancer cell lines examined, and it was the strongest in JHOC5 cells. Therefore, we chose JHOC5 cells for further analysis of Par3 function in ovarian cancer. Subcellular fractionation assays showed that Par3 protein was present in the nucleus, as well as the cytoplasm and membranes (Fig. 2b). Expression was particularly strong in the cytoplasm and nucleus of JHOC5 cells. Immunofluorescence staining revealed that Par3 localizes in the nucleus, cytoplasm, and focally at the membrane in JHOC5 cells (Fig. 2c). HaCaT cells, a normal immortalized keratinocyte line, were used as a control because these cells make normal cell contacts. As shown in Fig. 2c, nuclear localization of Par3 was clearly seen in JHOC5 cells, but was not detectable in HaCaT cells, consistent with a previous study [26]. These results suggested that Par3 was mislocalized in ovarian cancer cells.

### Par3 promotes invasion and cell proliferation ability in JHOC5 cells

**Fig. 3:**
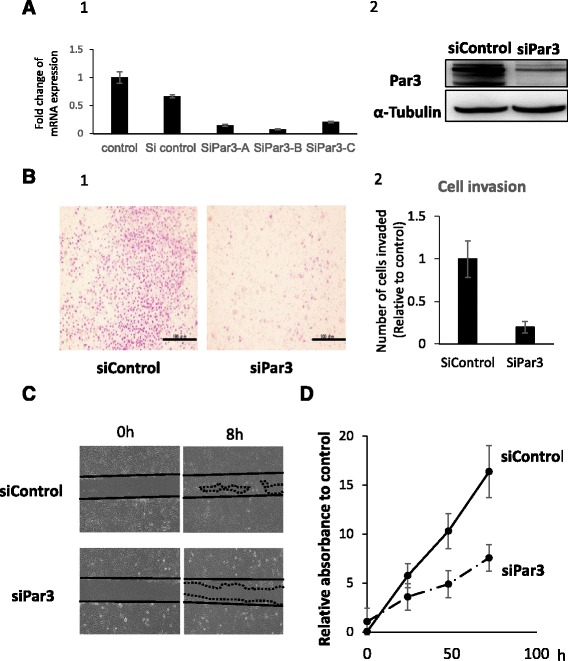
Downregulation of Par3 suppressed invasion and proliferation of JHOC5 cells. **a** Effect of siRNA against Par3. JHOC5 cells were transfected with Stealth RNAi against PAR3 (A: HSS125534, B: HSS183488, C: HSS183489) or control siRNA for 48 h. Total RNA was reverse transcribed and *PARD3* mRNA levels (Par3) were measured by a quantitative reverse transcription polymerase chain reaction. Expression was normalized to the expression of glyceraldehyde 3-phosphate dehydrogenase (GAPDH). Data are the mean (±SEM) of three independent experiments (A-1). siPar3-B was chosen for further analysis. Cells were transfected with siControl or siPar3, then 48 h after transfection, Par3 and α-Tubulin expression was analyzed by western blotting. The experiments were repeated at least 3 times (A-2). **b** Invasion assay. JHOC5 cells were transfected with the Par3 siRNA (siPar3) or control siRNA (siControl). Transfected cells were seeded in a Matrigel-coated Boyden chamber 48 h after transfection, and were allowed to invade for 24 h. Matrigel membranes were observed with an optical microscope. Scale bar indicates 100 μm (B-1). Numbers of cells invaded through matrigels were counted. Data are the mean (±SEM) of five different microscopic fields. The data is the representative of three independent experiments (B-2). **c**) Wound healing assay. JHOC5 cells were transfected with the Par3 siRNA (siPar3) or control siRNA (siControl), seeded onto 6-well culture plates, and grown as a monolayer for 48–60 h until 100% confluent. A scratch assay was then performed. Images of the same area of the wound were taken after 8 h to measure the width of the wound using fluorescence microscope. The data is the representative of three independent experiments. **d** Cell proliferation assay. To analyze the effect of Par3 knockdown on cell proliferation, 5000 cells were seeded onto 96-well plates 48 h after siRNA transfection. Cell Counting Kit-8 (Sigma Aldrich) was used to examine proliferation at 24, 48 and 72 h. Data are the mean (±SEM) of three wells. The data shown is representative of three independent experiments

Next, we investigated whether Par3 has a role in migration, invasion, and cell proliferation in JHOC5 cells. To accomplish this, we knocked down Par3 using siRNA. JHOC5 cells were transfected with three different siRNAs against Par3. qPCR analysis showed that siRNA B seemed to be the most effective in knocking down Par3 (Fig. 3A-1), therefore, we decided to use it for further studies. siPar3 B also sufficiently knocked down the Par3 protein level, as determined by western blot (Fig. 3A-2).

Invasion assays were performed using a Matrigel Invasion assay kit. In this study, cells invaded through an extracellular matrix barrier using 10% FBS as a chemoattractant, and cell invasion was assessed after 24 h. JHOC5 cells showed decreased invasive ability following siRNA knockdown of Par3 (Fig. 3B-1,2). In a wound healing assay, siPar3-treated JHOC5 cells showed delayed cell migration (Fig. 3c). Furthermore, in the cell proliferation assay, Par3 knockdown suppressed JHOC5 cell proliferation (Fig. 3d). The effect of Par3 knockdown was confirmed by western blotting at 24, 48,72 h (Additional file 1: Figure S1).

### Par3 affects IL-6 and STAT3 activation in JHOC5 cells

**Fig. 4:**
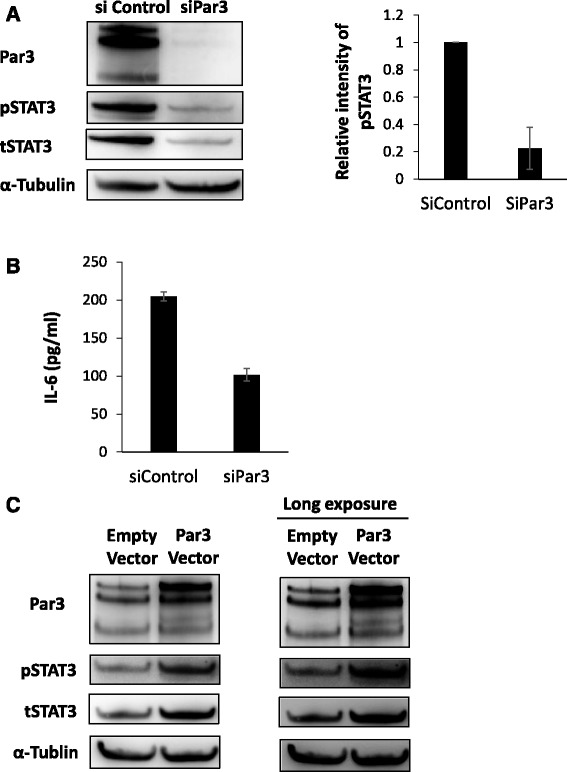
Par3 regulates IL-6/STAT3 pathway. **a** Effect of siPar3 on STAT3 expression. JHOC cells were transfected with siPAR3 or control siRNA (siControl). Total cell extracts were then obtained after 48 h, and phospho-STAT3, total STAT3, and α-Tubulin proteins were detected by western blotting. The right-hand histogram shows the relative intensities of the blots of pSTAT3 normalized to α-Tubulin. Data are the mean (±SEM) of three independent experiments. **b** Effect of siPar3 on IL-6 secretion. JHOC5 cells were transfected with siPar3 or control siRNA for 48 h, then conditioned media were collected for ELISA analysis of IL-6. Data are the mean (±SEM) of three independent experiments. **c** Effect of Par3 overexpression on STAT3 expression. JHOC5 cells were transfected with pcDNA 3.1 (empty vector) or myc-Par3 for 24 h and Par3, phospho-STAT, total STAT, and α-Tubulin levels were analyzed by western blotting

**Fig. 5:**
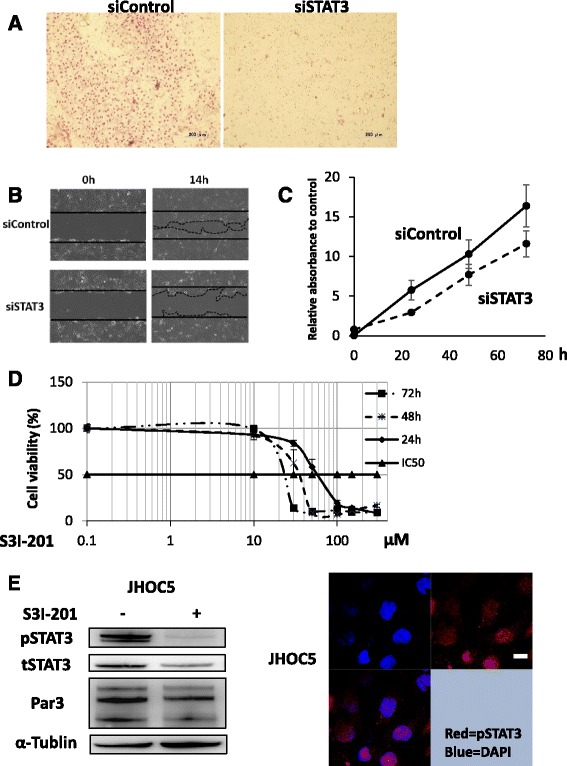
STAT3 regulates Par3 expression. **a** Invasion assay. JHOC5 cells were transfected with the STAT3 siRNA (siSTAT3) or control siRNA (siControl). The transfected cells were placed in a Matrigel-coated Boyden chamber 48 h after transfection, and allowed to invade for 24 h. Matrigel membranes were observed with an optical microscope. Scale bar indicates 200 μm. **b** Wound healing assay. JHOC5 cells were transfected with STAT3 siRNA (siSTAT3) or control siRNA (siControl), seeded onto 6-well culture plates, and grown as a monolayer for 48–60 h until 100% confluent. A scratch assay was then performed. Images of the same area of the wound were taken after 14 h to measure the width of the wound using fluorescence microscope. Data is representative of three independent experiments. **c** Cell proliferation assay. To analyze the effect of STAT3 knockdown on cell proliferation, 5000 cells were seeded onto 96-well plates 48 h after siRNA transfection. A Cell Counting Kit-8 (Sigma Aldrich) was used to examine proliferation. Data are the mean (±SEM) of four wells. The data shown is representative of three independent experiments. **d** Effect of STAT3 inhibition on JHOC5 cell proliferation. 5000 cells were seeded onto 96-well plates and incubated for 24 h. Then, they were grown with or without a STAT3 inhibitor (S3I-201) at the doses indicated. Cell proliferation was determined after 24, 48, and 72 h, using a Cell Counting Kit-8 (Sigma Aldrich). Data are the mean (±SEM) of four wells. Showing data is representative of two independent experiments. **e** Effect of STAT3 inhibition on Par3 expression. JHOC5 cells were treated with or without S3I-201 (100 μM) for 48 h. Cells were lysed and then phospho-STAT3, total STAT3, Par3, and α-Tubulin protein levels were detected by western blotting. **f** Immunofluorescent analysis of pSTAT3 expression. JHOC5 cells were grown on coverslips and then fixed and stained with anti-phospho-STAT3 and DAPI. Scale bar indicates 10 μm

Having shown that Par3 is involved in promoting cell proliferation, migration, and invasion, we were interested in further investigating its molecular mechanism. To do this, we focused on the IL-6/STAT3 pathway. Western blot analysis revealed that STAT3 activation (phosphorylated STAT3 (Tyr705)) decreased upon the loss of Par3 in JHOC5 cells (Fig. 4a). ELISA analysis demonstrated that siPar3 also significantly decreased IL-6 secretion by JHOC5 cells (Fig. 4b). Based on these results, we reasoned that the IL-6/STAT3 pathway might be important for regulating Par3 function in certain types of ovarian cancer cells. To confirm whether Par3 regulates the STAT3 pathway, we analyzed the effect of wild type Par3 overexpression on STAT3 activation. As expected, Par3 upregulated the level of phospho-STAT3 in JHOC5 cells (Fig. 4c). Furthermore, analysis of siSTAT3-treated JHOC5 cells in cell invasion assays, wound healing assays, and cell proliferation assays confirmed the STAT3-dependence of JHOC cells in these assays (Fig. 5a-c). In addition, treatment with a STAT3 inhibitor (S3I-201: SC-204304) suppressed cell proliferation (Fig. 5d). Taken together, these results indicated that JHOC5 cell invasion, proliferation, and migration were dependent on the STAT3 pathway.

We also determined whether STAT3 could regulate Par3 expression; western blot analysis showed that Par3 expression was downregulated in the presence of STAT3 inhibitor (S3I-201) in JHOC5 cells (Fig. 5e).

Furthermore, immunofluorescence analysis showed that active STAT3 (pSTAT3) localizes to the nucleus in JHOC cells (Fig. 5f). As we demonstrated in Fig. 2c, Par3 localizes to both the nucleus and cytoplasm in JHOC5 cells. Taken together, these results implied that Par3 interacts either directly or indirectly with active STAT3 and IL-6, suggesting that Par3 may regulate cellular invasion and proliferation in JHOC5 cells via the IL-6/STAT3 pathway.

## Discussion

In the present study, we observed that high Par3 expression was significantly associated with advanced stage and peritoneal dissemination at diagnosis. Furthermore, low Par3 expression was associated with good prognosis. We also showed that Par3 promotes invasive properties in JHOC5 cells through the IL-6/STAT3 pathway.

Previous studies have reported diverse functions of Par3, including regulation of cell proliferation, cell polarity, cell migration, and cell invasion in a variety of different cancer cell types. Recently, polarity gene disruption has been observed in a subset of human cancers using genome-wide screening strategies like high-resolution copy number array analysis [30]. The results indicate that dysregulation of cell polarity affects cancer progression. To date, disruption of polarity complexes including Scribble, Par3, and Crumbs complexes has been considered essential for cancer development [7, 12, 38–40]. Among these complexes, the Par3 complex is thought to be a master regulator controlling ubiquitous functions [41, 42]. Considering the role of Par3 in carcinogenesis, it has recently been shown that depletion of Par3 along with the expression of oncogenic Notch and Ras in murine mammary gland cells is associated with a tumor-promoting effect and metastasis [22]. Moreover, Par3 inactivation was discovered in 8% of squamous cell lung cancers and Par3 immunohistochemical analysis in lung cancers also demonstrated its contribution to cancer development [25]. In contrast, a study of skin tumorigenesis demonstrated that Par3 deficiency in mice resulted in a predisposition toward keratoacanthomas, a common low-grade skin tumor from various cellular origins [24]. Other studies reported that Par3 overexpression was associated with cancer initiation [27]. All these results indicate that Par3 can play various roles in regulating tumor formation.

Previous studies implied that Par3 disruption in squamous cell carcinomas and glioblastomas was caused by mutations of the ﻿*PARD3* gene [30]. However, according to TCGA data [43], only one such mutation was detected in 316 cases of ovarian serous adenocarcinoma. These conflicting observations in various cancers including ovarian cancer make it difficult to investigate Par3 function.

In this study, microarray analysis of 50 ovarian cancer cases indicated that low Par3 expression was associated with good prognosis (Fig. 1). We also observed that Par3 might be mislocalized to the cytoplasm and the nucleus (Fig. 2b and c). Furthermore, Par3 expression promotes cell invasion, migration, and cell proliferation in JHOC5 cells (Fig. 3b-d). We investigated the underlying mechanism of these Par3 functions by focusing on the IL-6/STAT3 pathway. Par3 knockdown suppressed STAT3 activation and IL-6 levels (Fig. 4a, b). Therefore, Par3 may exert its oncogenic potential through the STAT3 pathway in a subset of ovarian cancer cells that are similar to JHOC5 cells. Although at present, we have not been able to investigate the mechanism by which Par3 regulates the IL-6/STAT3 pathway, previous studies have shown that the Par3 complex has a strong relationship with the STAT3-IL-6 axis in mammalian cells [32]. Here, we provide the first indication that Par3 is associated with ovarian cancer progression through the IL-6/STAT3 pathway.

Clearly, this ovarian cancer model is limited, since we have not been able to compare the function and localization of Par3 to normal ovarian cells. The origin of ovarian cancer is controversial [43] which makes it difficult to define “normal” ovarian cell lines compared with malignant cell lines, although ovarian surface epithelial (OSE) cells have been used as a model to study ovarian carcinogenesis. As an alternative, we used HaCaT cells, immortalized human keratinocytes, to see where Par3 is normally localized. Intriguingly, as seen in Fig. 2c, we found that Par3 is expressed in the nucleus as well as the cytoplasm in JHOC5 cells. As far as we know, this is the first observation of nuclear Par3 expression in ovarian cancer cells.

Though the exact mechanism through which Par3 affects tumor formation remains to be investigated, our observations may explain how Par3 may be involved in tumor malignancy, which could be largely dependent on the reconstitution of STAT3 signaling in ovarian cancer. Recently, it was reported that aberrant activation of the STAT3 pathway is found in more than 70% of ovarian cancers and was associated with decreased OS [44]. Moreover, therapeutic strategies targeting STAT3 signaling are being developed [45]. In terms of developing prognostic biomarkers, our study suggests that Par3 could be a candidate for ovarian cancer management, especially in monitoring STAT3 signaling in metastatic ovarian cancer.

## Conclusions

This study highlights the association between low Par3 expression and good prognosis. It also showed Par3 expression is likely involved in ovarian cancer progression, especially in peritoneal metastasis. The underlying mechanism may be that Par3 modulates IL-6 /STAT3 signaling. Par3 could be a candidate for prognostic biomarkers in ovarian cancer in monitoring STAT3 signaling.

## Additional file

Additional file 1: Figure S1. Par3 is sufficiently knocked down at 24, 48, and 72 h. JHOC cells were transfected with siPar3 or control siRNA (siControl). Total cell extracts were then taken after 24, 48, and 72 h. Par3 and α-Tubulin protein were detected by western blotting. (PPTX 139 kb)

## Abbreviations

aPKC: Atypical protein kinase C
ELISA: Enzyme-linked immune-sorbent assay
EMT: Epithelial mesenchymal transition
IL-6: Interleukin-6
JNK: c-Jun N-terminal kinases
OS: Overall survival
Par 3: Partitioning defective 3
PFS: Progression free survival
qPCR: Quantitative polymerase chain reaction
STAT3: Signal tranducer and activator of transcription 3.
