# Supplementary figures and images for: Expression of Par3 polarity protein correlates with poor prognosis in ovarian cancer
Source: BMC Cancer. 2016 Nov 17;16:897. doi: 10.1186/s12885-016-2929-2 (PMC5114836; doi:10.1186/s12885-016-2929-2)

## Slide 1
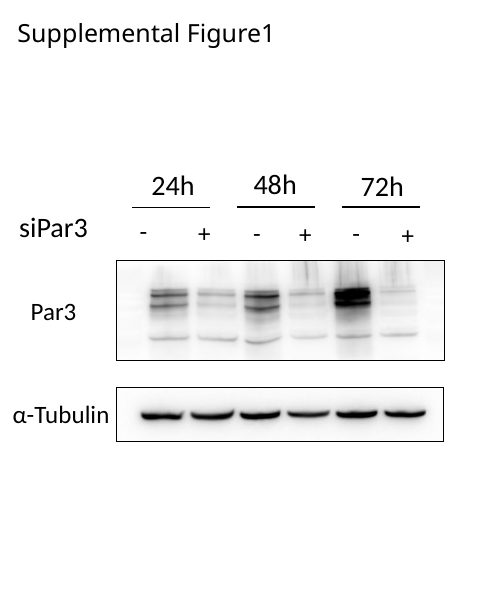

Supplemental Figure1
48h
24h
72h
siPar3
-
+
-
-
+
+
Par3
α-Tubulin

Supplement: Additional file 1: Figure S1. — Par3 is sufficiently knocked down at 24, 48, and 72 h. JHOC cells were transfected with siPar3 or control siRNA (siControl). Total cell extracts were then taken after 24, 48, and 72 h. Par3 and α-Tubulin protein were detected by western blotting. (PPTX 139 kb) [file 12885_2016_2929_MOESM1_ESM.pptx]
